# Supplementary material for: Neutral Forces and Balancing Selection Interplay to Shape the Major Histocompatibility Complex Spatial Patterns in the Striped Hamster in Inner Mongolia: Suggestive of Broad-Scale Local Adaptation
Source: Genes (Basel). 2023 Jul 22;14(7):1500. doi: 10.3390/genes14071500 (PMC10379431; doi:10.3390/genes14071500)
Supplement: Supplementary file 1 [file genes-14-01500-s001.zip › genes-2497238-supplementary.pdf]

Table S1. Sampling information of different populations of striped hamster

Pop	Sites	Longitude	Latitude	Elevation(m)	Samples
N1	New Barag Right Banner, Hulun Buir	116.85ºE	48.81ºN	552	3
N2	East Ujimqin Banner, Xilin Gol	116.59ºE	45.60ºN	916	17
N3	Qog UI, Xilin Gol	115.92ºE	44.18ºN	947	24
N4	Baiyin Siler, Xilin Gol	116.78ºE	44.08ºN	1215	1
W2	Abag Banner, Xilin Gol	114.97ºE	44.03ºN	1144	62
W4	West Ujimqin Banner, Xilin Gol	116.73ºE	44.59ºN	968	111
W5	Horqin Left Middle Banner, Tongliao	121.55ºE	44.27ºN	203	4

Table S2. Characteristics of 7 microsatellite loci of striped hamster

Loci	Primer Sequence (5'→3')	Allele Size (bp)	Genbank ID
CHM93	F: TCTGTGTGTCTGTGAATGCG<br>R: GGATGTAAGATGGCTCCGAA	276	FJ426316
CHM108	F: CAATGGCTGCTAAGAGAGGG<br>R: GTTCTTTCCCACTCACAATTTTCCTTC	276	FJ426318
CHM120	F: GATAGGAGGGAGCAAAAGGG<br>R: CCTGAGAGCCTCAGAGCAGT	242	FJ426320
CHM124	F: CAGATGCCCAAACTCTCTCC<br>R: GTTCTTGTCACCCCATGGACTACA	284	FJ426321
CHM140	F: GCAGCTGGTTTTGAGCTACC<br>R: GTTCTTTGTTTGATCACTGGAACCCA	250	FJ426323
CHM147	F: CATCTGGGCTTTCAATGGAT<br>R: GCTCCCTAAATAACCCCCAA	241	FJ426324
SSR29	F: CAGGGAAATGATGATACC<br>R: TCAGCAGCACTCAGAAGG	357	EF530613

Table S3. Eleven flea species and nineteen gamasid mite species detected in this study

No.	Flea species	Gamasid Mite Species
1	*Citellophilus tesquorum*	*Eulaclaps novus*
2	*Neopsylla bidentatiformis*	*Eulaclaps cricetuli*
3	*Neopsylla pleskei*	*Haemogamasus kusumotoi*
4	*Frontopsylla luculenta*	*Haemogamasus mandschuricus*
5	*Rhadinopsylla dives*	*Haemogamasus kitanoi*
6	*Stenoponia formozovi*	*Hirstionyssus transiliensis neimongkuensis*
7	*Amphipsylla longispina*	*Haemolalaps glasgowi*
8	*Amphipsylla primaris*	*Haemolalaps semidestertus*
9	*Ophthalmopsylla kukuschkini*	*Hypoaspis miles*
10	*Nosopsylla laeviceps*	*Hypoaspis pavlovski*
11	*Unident_flea* [a]	*Hypoaspis lubrica*
12	/	*Macrocheles decoloratus*
13	/	*Macrocheles matrius*

14	/	*Poecilochirus necrophori*
15	/	*Poecilochirus davydovae*
16	/	*Parasitus* sp.1[b]
17	/	*Parasitus* sp.2 [b]
18	/	*Parasitus* sp.3 [b]
19	/	*Unident_gamasid_mite* [a]

[a] impossible to determine its taxonomic classification due to specimen damage;

[b] Undetermined species of the genus *Parasitus*

Table S4. Results from maximum likelihood codon-based models of selection using EastyCodeml.

Model	np	Ln L	Parameter estimates	Model compared	LRT P-value
M3	164	-2014	p_0 = 0.583, p_1 = 0.274, p_2 = 0.143, ω_0 = 0.063, ω_1 = 0.794, ω_2= 2.145	M0 vs. M3	P < 0.0001
M0	160	-2164	ω_0 = 0.436		
M2a	163	-2014	p_0 = 0.596, p_1 = 0.328, p_2 = 0.077, ω_0 = 0.069 , ω_1 = 1 , ω_2 = 3.081	M1a vs. M2a	P < 0.0001
M1a	161	-2027	p_0 = 0.613, p_1 = 0.387, ω_0 = 0.062 , ω_1 = 1		
M8	163	-2012	p_0 = 0.87, p_1 = 0.130, p = 0.214, q = 0.441, ω = 2.215	M7 vs. M8	P < 0.0001
M7	161	-2023	p = 0.064, q = 0.080		
M8a	162	-2020	p_0 = 0.732, p_1 = 0.268 p = 0.390, q = 2.177, ω = 1	M8a vs. M8	P < 0.0001

Abbreviation: ω = selection parameter; p_n = proportion of sites that fall into the ω_n site class; p, q = shape parameters of the β function (for models M7 and M8).
